# Supplementary material for: Frictional internal work of damped limbs oscillation in human locomotion
Source: Proc Biol Sci. 2020 Jul 29;287(1931):20201410. doi: 10.1098/rspb.2020.1410 (PMC7423663; doi:10.1098/rspb.2020.1410)
Supplement: Supplementary Materials and Methods [file rspb20201410supp1.pdf]

Supplementary materials to accompany the article:

FRICTIONAL INTERNAL WORK OF DAMPED LIMBS OSCILLATION IN HUMAN LOCOMOTION

Alberto E. Minetti, Alex P. Moorhead and Gaspare Pavei

In Proceedings of the Royal Society B: doi:10.1098/rspb.2020.1410

(equation numbering in bold refers to those reported also in the main text)

The theory

The following deals with the dynamics of a pendulum affected only by gravity and by a viscous damping torque: the first part will illustrate the mathematical solution for (small) oscillations of a straight pendulum (angle θ with the vertical axis as zero when fulcrum is at the top, positive values when turning counterclockwise), the second part will consider an inverted pendulum (angle θ is zero with fulcrum at the bottom, positive values when turning clockwise). Although a single differential equation would manage both situations, a simplification introduced to solve it (see below) forces splitting the problem into two separate cases. The following solutions model the behaviour of a passive straight pendulum oscillating from a manually imposed initial angle, and of a passive inverted pendulum moving upward after a manually provided impulsive push (see Experimental protocol).

Damped Straight Pendulum

In a viscously damped (b, N·m·s·rad^{-1}) pendulum of mass m (and massless rod) located at a distance R from the pivoting point, because of the Moment Conservation Law:

$$I\ddot{\theta} = \sum T \qquad (1, \mathbf{2.1})$$

where the moment of inertia, gravitational and damping torques are:

$$I = m\,R^2, \quad T_g = -R\,m\,g\,sin\,\theta \quad \text{and} \quad T_d = -b\,\dot{\theta} \qquad (2)$$

When the rod also has a mass, equations (2) become

$$I = m\,R_g^2, \quad T_g = -R\,m\,g\,sin\,\theta \quad \text{and} \quad T_d = -b\,\dot{\theta} \qquad (3)$$

where m is the total mass of the pendulum, with the centre of mass located at a distance R from the pivot, and R_g is the radius of gyration about the pivot of the pendulum.

Thus

$$\ddot{\theta} = -\frac{b}{m\,R_g^2}\dot{\theta} - g\frac{R}{R_g^2}\,sin\,\theta \qquad (4)$$

and, by assuming $sin\,\theta \approx \theta$, with the restriction $-0.7 < \theta < +0.7$, corresponding to $-40^o < \theta < +40^o$ (with a mean approximation of 2.81%),

$$\ddot{\theta} = -\frac{b}{m\,R_g^2}\dot{\theta} - g\frac{R}{R_g^2}\,\theta \qquad (5, \mathbf{2.2})$$

This is a 2nd Order Linear Ordinary Differential Equation (ODE) of the form

$$\ddot{\theta} + A\dot{\theta} + B\theta = 0 \qquad (6)$$

where

$$A = \frac{b}{m\,R_g^2} \quad \text{and} \quad B = g\frac{R}{R_g^2} \qquad (7)$$

By substituting $\theta = e^{r\,t}$ into equation (6), we obtain

$$r^2 e^{r\,t} + A\,r\,e^{r\,t} + B\,e^{r\,t} = 0 \qquad (8)$$

or, as $e^{r\,t}$ is always > 0,

$$r^2 + A\,r + B = 0 \qquad (9)$$

where solutions are

$$r_{1,2} = \frac{-A \pm \sqrt{A^2 - 4B}}{2} \qquad (10)$$

The general solution of the 2nd Order Linear ODE (eq. (6)) is

$$\theta(t) = C_1\,e^{r_1\,t} + C_2\,e^{r_2\,t} \qquad (11, \mathbf{2.3})$$

where $C_1$and C_2 will be set according to the initial conditions of the pendulum in terms of θ (rad) and $\dot{\theta}$ (rad/s). Depending on $A^2 - 4B = \rho$, pendulum oscillations are overdamped ($\rho > 0$), critically damped ($\rho = 0$), or underdamped ($\rho < 0$).

Our joint friction experiment is supposed to provide underdamped oscillations, as suggested when observing the upper or lower limb passively behaving as a straight pendulum).

Thus

$$r_{1,2} = \frac{-A \pm i\sqrt{4B - A^2}}{2} \quad (12)$$

and

$$\theta(t) = C_1\, e^{\frac{-A + i\sqrt{4B-A^2}}{2}t} + C_2\, e^{\frac{-A - i\sqrt{4B-A^2}}{2}t}$$

$$\theta(t) = e^{-\frac{A}{2}t}\left(C_1\, e^{i\frac{\sqrt{4B-A^2}}{2}t} + C_2\, e^{-i\frac{\sqrt{4B-A^2}}{2}t}\right) \quad (13)$$

But, due to Euler's Identity $e^{i\,\omega\,t} = cos(\omega\, t) + i\, sin(\omega\, t)$ and

$e^{-i\,\omega\,t} = cos(\omega\, t) - i\, sin(\omega\, t)$, and with $\omega = \frac{\sqrt{4B-A^2}}{2}$

$$\theta(t) = e^{-\frac{A}{2}t}\left(C_1\left(cos(\omega\, t) + i\, sin(\omega\, t)\right) + C_2\left(cos(\omega\, t) - i\, sin(\omega\, t)\right)\right) \quad (14)$$

Then

$$\theta(t) = e^{-\frac{A}{2}t}\left((C_1 + C_2)cos(\omega\, t) + i(C_1 - C_2)sin(\omega\, t)\right) \quad (15)$$

or, by assimilating $C_1 + C_2$ and $i(C_1 - C_2)$ to the general constant K_1 and K_2, respectively:

$$\theta(t) = e^{-\frac{A}{2}t}\left(K_1\, cos(\omega\, t) + K_2\, sin(\omega\, t)\right) \quad (16)$$

from which, by imposing initial conditions in terms of angle θ_0 and angular speed $\dot{\theta}_0$, Cauchy Problem (of initial values θ_0 and $\dot{\theta}_0$) can be solved as:

$$\theta(0) = K_1 = \theta_0 \quad (17)$$

and

$\dot{\theta}(t)$

$$= -\frac{A}{2} e^{-\frac{A}{2}t}\left(K_1\, cos(\omega\, t) + K_2\, sin(\omega\, t)\right) + e^{-\frac{A}{2}t}\left(\omega\, K_2\, cos(\omega\, t) - \omega\, K_1\, sin(\omega\, t)\right)$$

$$\dot{\theta}(0) = \omega\, K_2 - \frac{A\, K_1}{2} = \dot{\theta}_0$$

leading to

$$K_2 = \frac{2\,\dot{\theta}_0 + A\,\theta_0}{2\omega} \qquad (18)$$

Pendulum angle time course, $\theta(t)$ (eq. (16)), corresponds to

$$\theta(t) = K\, e^{-\frac{A}{2}t}\, sin(\omega\, t\ +\ \phi) \qquad (19,\ \mathbf{2.4})$$

where

$$K = \sqrt{K_1^2 + K_2^2} \quad \text{and} \quad \phi = \pi\, sgn(sgn(K_2) - 1) + arctan\frac{K_1}{K_2} \qquad (20)$$

or

$$K = sgn\,(K_2)\sqrt{K_1^2 + K_2^2} \quad \text{and} \quad \phi = arctan\frac{K_1}{K_2} \qquad (21)$$

Equation (19), in extended form, is

$$\theta(t) = 2\, R_g \sqrt{m\frac{g\, R\, m\, \theta_0^2 + R_g^2\, m\, \dot{\theta}_0^2 + \theta_0\, \dot{\theta}_0\, b}{4\, g\, R\, m^2 R_g^2 - b^2}}\; e^{-\frac{b}{2\, m\, R_g^2}t}\; sin\left(\frac{\sqrt{4\, g\, R\, m^2 R_g^2 - b^2}}{2\, m\, R_g^2}\, t + \right.$$

$$\left. \pi\, sgn\left[sgn(\frac{2\, R_g^2\, m\, \dot{\theta}_0 + \theta_0\, b}{\sqrt{4\, g\, R\, m^2 R_g^2 - b^2}}) - 1\right] + arctan\left(\theta_0 \frac{\sqrt{4\, g\, R\, m^2 R_g^2 - b^2}}{2\, R_g^2\, m\, \dot{\theta}_0 + \theta_0\, b}\right)\right) \qquad (22)$$

or

$$\theta(t) = 2\, R_g\, sgn(\frac{2\, R_g^2\, m\, \dot{\theta}_0 + \theta_0\, b}{\sqrt{4\, g\, R\, m^2 R_g^2 - b^2}}) \sqrt{m\frac{g\, R\, m\, \theta_0^2 +\ R_g^2\, m\, \dot{\theta}_0^2 + \theta_0\, \dot{\theta}_0\, b}{4\, g\, R\, m^2 R_g^2 - b^2}}$$

$$e^{-\frac{b}{2\,m\,R_g^2}t}\, sin\left(\frac{\sqrt{4\,g\,R\,m^2 R_g^2 - b^2}}{2\,m\,R_g^2}\,t + arctan\,\theta_0 \frac{\sqrt{4\,g\,R\,m^2 R_g^2 - b^2}}{2\,R_g^2\,m\,\dot{\theta}_0 + \theta_0\,b}\right) \qquad (23)$$

In these solution equations, the damping factor b is the only unknown variable as all the other symbols, except from the ones related to initial conditions (θ_0 , $\dot{\theta}_0$), are set by the anatomical characteristics of each subject and the inertial property of the limb (+load for the inverted pendulum, see below).

From the comparison between the experimental and predicted time courses of lower limb oscillations, iteratively obtained for a wide range of b values, the best estimation of damping could be obtained for each experimental condition. An alternative, of similar complexity, is a non-linear custom regression (eq. 22), fed with the data of the time course of pendulum oscillation, where the damping coefficient b could be estimated.

The easiest procedure is to extract from experimental oscillations the pendulum angles (y) and timing (t) of the repeated swing inversions, rectify them (see Figure 3) and perform an exponential regression (the 'true' one (e.g. Labview, National Instruments, US or Grapher, Apple Computers, US), not the linearized version (Excel, Microsoft, US), see below), in the form

$$y = p\,e^{qt} \qquad (24)$$

which would estimate the term q ($= -\frac{A}{2}$ of eq. (22)) from which b can be obtained as

$$b = -2\,m\,R_g^2\,q\,. \qquad (25, \mathbf{2.5})$$

Damped Inverted Pendulum

The above solution for the straight pendulum cannot be used for the inverted pendulum because the assumption $sin\,\theta \approx \theta$ does not hold for angles with operative range around π. To use the same simplification, reference angle for zero should be in the middle of its new operative range, i.e. when the pendulum is at the top of its trajectory. We also introduced positive angle when moving clockwise.

Equations (2) and (3) should be changed into

$$I = m\,R^2, \quad T_g = R\,m\,g\,sin\,\theta \quad \text{and} \quad T_d = -b\,\dot{\theta} \tag{26}$$

and

$$I = m\,R_g^2, \quad T_g = R\,m\,g\,sin\,\theta \quad \text{and} \quad T_d = -b\,\dot{\theta} \tag{27}$$

The successive equations for the straight pendulum still hold, up to eq. (11), if

$$A = \frac{b}{m\,R_g^2} \quad \text{and} \quad B = -g\frac{R}{R_g^2} \tag{28}$$

Now $\rho = A^2 - 4B$ is always positive and, although improper for describing the travel of a damped inverted pendulum from side to side while passing through the top point, the dynamics gets closer to an overdamped condition, which is associated to a different, much simpler solution.

In this case the solution of the 2nd Order Linear ODE (eq. (6) and (9)) is:

$$\theta(t) = C_1\,e^{M\,t} + C_2\,e^{N\,t} \qquad (29,\ \mathbf{2.6})$$

where

$$M = \frac{-A+\sqrt{A^2-4B}}{2} \quad \text{and} \quad N = \frac{-A-\sqrt{A^2-4B}}{2} \tag{30}$$

From differentiation of equation (29), angular speed can be obtained as:

$$\dot{\theta}(t) = C_1\,M\,e^{M\,t} + C_2\,N\,e^{N\,t} \tag{31}$$

Equations (29) and (31) allow to estimate the value of $C_1$and C_2according to the initial conditions $\theta_0 = \theta(0)$ and $\dot{\theta}_0 = \dot{\theta}(0)$:

$$C_1 = \frac{\dot{\theta}_0 - N\theta_0}{M-N} \quad \text{and} \quad C_2 = -\frac{\dot{\theta}_0 - M\theta_0}{M-N} \tag{32}$$

leading to the closed form of equations (29) and (31):

$$\theta(t) = \frac{(\dot{\theta}_0 - N\theta_0)\,e^{M\,t} - (\dot{\theta}_0 - M\theta_0)\,e^{N\,t}}{M-N} \tag{33}$$

$$\dot{\theta}(t) = \frac{(\dot{\theta}_0 - N\theta_0)\,M\,e^{M\,t} - (\dot{\theta}_0 - M\theta_0)\,N\,e^{N\,t}}{M-N} \tag{34}$$

Differently from the straight pendulum experiments, only one swing is obtained at a time from an inverted one, from which the damping factor b could be estimated.

Similarly to the previous case, though, a non-linear, custom regression could be used to estimate M and N, thus A and, consequently, b. Also, in this case, this process looked cumbersome and complex, and a different analysis strategy was developed.

Known data from the single experimental swing, within an angular range where the dynamics is not affected anymore by the operator's push and has not yet reached the stop boundary for the inverted pendulum, are the initial and final angle (θ_0 and θ_{end}, with respect to the vertical), the initial and final angular speed ($\dot{\theta}_0$ and $\dot{\theta}_{end}$), and the time duration of the oscillation from the initial angle to the final one (t_{swing}). A software routine has been designed (Labview, National Instruments, US), to generate a multitude of angle time courses (equation (33)) with a resolution of 0.1 ms, starting from the same initial conditions (θ_0 and $\dot{\theta}_0$), each of them depending on a given b value chosen from an extended, realistic and highly resoluted (step 0.001 N m s/ rad) range. The algorithm selected b values associated to time courses capable to reach both θ_{end} and $\dot{\theta}_{end}$ after a time t_{swing}. Thresholds for the allowed approximation of the three simultaneous goals were manually increased as to obtain a coefficient of variation of b ($CV_b = \frac{SD_b}{\bar{b}}$) smaller than 1%.

An additional method is to check at which b value the simulated oscillation reaches the minimum angular speed at the same time interval from the end of the push as found during experiments. By differentiating equation (34) and equating the result to zero, the timing of the minimum angular speed ($t_{\dot{\theta}_{MIN}}$) can be obtained as:

$$t_{\dot{\theta}_{MIN}} = ln\left(\sqrt[M-N]{\frac{(\dot{\theta}_0 - M\theta_0)N^2}{(\dot{\theta}_0 - N\theta_0)\, M^2}} \right) \tag{35}$$

or

$$t_{\dot{\theta}_{MIN}} = \frac{ln\left(\frac{\dot{\theta}_0 - M\theta_0}{\dot{\theta}_0 - N\theta_0}\right) + 2(ln\, N - ln\, M)}{M-N} \tag{36}$$

Similar thresholds were allowed to detect the reach of a close approximation with experimental $t_{\dot{\theta}_{MIN}}$.

Energy dissipated during a locomotory cycle, and the associated metabolic cost to overcome the damping of the limbs.

The mechanical energy necessary to maintain a periodic oscillatory movement

$$\theta = A_h \, sin(\omega \, t) \qquad (37, \textbf{2.7})$$

where A_h is half the angle range and ω is the frequency coefficient ($= 2 \, \pi \, f$, with f in Hz), of a damped pendulum with viscous friction b (J·m·s·rad^{-1}) is calculated by integrating over one cycle the work rate ($\dot{W}$, W) equation of energy dissipation.

Actually,

$$\dot{W} = T_d \, \dot{\theta} \qquad (38)$$

where the damping torque or moment (T_d, N·m) is

$$T_d = b \, \dot{\theta} \qquad (39)$$

and angular speed, from differentiation of eq. 37, is

$$\dot{\theta} = A_h \, \omega \, cos(\omega \, t) \qquad (40)$$

Then, the energy dissipated by the damping effect over one cycle (W_{1c}, J) is

$$W_{1c} = \int_0^{\frac{2\pi}{\omega}} \dot{W} \, dt = \int_0^{\frac{2\pi}{\omega}} b \, A_h^2 \, \omega^2 \, cos^2(\omega \, t) \, dt \qquad (41)$$

or

$$W_{1c} = \frac{1}{\omega} \int_0^{2\pi} b \, A_h^2 \, \omega^2 \, cos^2(\omega \, t) \, d(\omega \, t) = \pi \, b \, A_h^2 \, \omega \qquad (42)$$

In order to obtain an estimate of the mechanical cost of transport just due to the internal friction of a damped limb (C_{mif}, J m^{-1}), we need to calculate the distance travelled (d_{1c}, m) by a body with limbs behaving as described by eq. 37. By assuming, as in eq. 5, $sin \, \theta \approx \theta$, ($-0.7 < \theta < +0.7$ rad, or $-40^o < \theta < +40^o$), and a limb length R_L (m)

$$d_{1c} = 4 \, R_L \, sin(A_h) \approx 4 \, R_L \, A_h \qquad (43)$$

Average speed $\bar{v}$ (m s^{-1}) can be expressed as the distance travelled by limb extremity during half a cycle, divided by half the oscillation period:

$$\bar{v} = \frac{2\,R_L\,A_h}{1/(2\,f)} = 4\,R_L\,f A_h \qquad (44)$$

Thus, by combining eq. 42, 43 and 44, replacing ω and by including body mass (*m,* kg):

$$C_{\text{mif}} = \frac{\pi^2\,b}{8\,{R_L}^2}\bar{v} \qquad (45)$$

The estimate above refers to a single limb. The mass-specific **m**echanical cost of transport to overcome **i**nternal **f**riction of **a**ll the 4 limbs in the body (C_{mifa}, J kg^{-1} m^{-1}) is:

$$C_{\text{mifa}} = \frac{\pi^2\,B}{8\,m\,{R_L}^2}\bar{v} \qquad (46,\ \mathbf{2.8})$$

with $B = \sum_1^4 b_i$, where b_i are the damping coefficients of each proximal joint of the limbs. Eq. 46 assumes the 4 limbs of the same length (R_L) and spanning the same angle range ($2A_h$) during locomotion.
